# Improving efficacy of repetitive transcranial magnetic stimulation for treatment of Parkinson disease gait disorders

**DOI:** 10.3389/fnhum.2024.1445595

**Published:** 2024-08-26

**Authors:** Rupsha Panda, Joseph A. Deluisi, Taraz G. Lee, Sheeba Davis, Isabel Muñoz-Orozco, Roger L. Albin, Michael Vesia

**Affiliations:** ^1^Department of Psychology, University of Michigan, Ann Arbor, MI, United States; ^2^School of Kinesiology, University of Michigan, Ann Arbor, MI, United States; ^3^Department of Neurology, University of Michigan, Ann Arbor, MI, United States; ^4^Neurology Service & GRECC, VAAAHS, Ann Arbor, MI, United States

**Keywords:** Parkinson disease, transcranial magnetic stimulation, plasticity, freezing of gait, cerebellum, prefrontal cortex, parietal cortex

## Abstract

Parkinson disease (PD) is a neurodegenerative disorder that causes motor and cognitive deficits, presenting complex challenges for therapeutic interventions. Repetitive transcranial magnetic stimulation (rTMS) is a type of neuromodulation that can produce plastic changes in neural activity. rTMS has been trialed as a therapy to treat motor and non-motor symptoms in persons with Parkinson disease (PwP), particularly treatment-refractory postural instability and gait difficulties such as Freezing of Gait (FoG), but clinical outcomes have been variable. We suggest improving rTMS neuromodulation therapy for balance and gait abnormalities in PwP by targeting brain regions in cognitive-motor control networks. rTMS studies in PwP often targeted motor targets such as the primary motor cortex (M1) or supplementary motor area (SMA), overlooking network interactions involved in posture-gait control disorders. We propose a shift in focus toward alternative stimulation targets in basal ganglia-cortex-cerebellum networks involved in posture-gait control, emphasizing the dorsolateral prefrontal cortex (dlPFC), cerebellum (CB), and posterior parietal cortex (PPC) as potential targets. rTMS might also be more effective if administered during behavioral tasks designed to activate posture-gait control networks during stimulation. Optimizing stimulation parameters such as dosage and frequency as used clinically for the treatment of depression may also be useful. A network-level perspective suggests new directions for exploring optimal rTMS targets and parameters to maximize neural plasticity to treat postural instabilities and gait difficulties in PwP.

## Introduction

Parkinson disease (PD) is a common neurodegenerative disorder affecting motor and cognitive functions (Albin, 2023). PD is a significant cause of disability in the aging population. Distal limb bradykinesia is the defining symptom of the disease, accompanied by rigidity, tremors, and balance and gait disturbances (Davie, 2008; Bohnen et al., 2022b; Bologna et al., 2023). Although PD is historically described as a motor disorder, it is a complex cognitive-motor disorder (Sarter et al., 2021). In line with the diverse array of motor and cognitive symptoms associated with PD, current treatments take many forms. Modern therapies include medications such as dopamine replacement therapy, surgical interventions like deep brain stimulation, and assistive treatments (e.g., physical, occupational, or speech therapies) aimed at improving motor and non-motor features. Although existing treatments frequently affect significant improvements, important clinical features are refractory to treatment. Treatment refractory postural instability and gait disorders are common and morbid features of advancing PD. Even when existing treatments are effective, there may be complicated side effects. Chronic use of dopaminergic medications can lead to behavioral problems and is associated with motor fluctuations and troublesome involuntary movements. Surgical therapies, such as deep brain stimulation, improve motor features, but only a limited number of persons with Parkinson (PwP) are eligible, and mood disorders are a common side effect (Skidmore et al., 2006; Morgante et al., 2007).

Given the shortcomings of existing treatments, there has been increased interest recently in repetitive transcranial magnetic stimulation (rTMS) as a neuromodulation therapy for PD. Since rTMS is non-invasive, it may present a safer alternative treatment to invasive surgeries. rTMS is considered to be more precise than existing medications, as it targets regions in the brain that are interconnected both structurally and functionally through direct and multisynaptic anatomical connections (Siebner et al., 2022). rTMS causes changes in neuronal excitability akin to long-term potentiation or long-term depression, depending on which protocols are employed and where stimulation is targeted. However, current rTMS protocols show inconsistent efficacy as a treatment for balance and gait abnormalities in PwP (Chung and Mak, 2016; Xie et al., 2021).

In this opinion piece, we offer suggestions for increasing rTMS treatment efficacy for balance and gait disorders in PwP. Most prior investigations of rTMS for motor deficits in PD targeted motor targets such as the primary motor cortex (M1) or supplementary motor area (SMA; Xie et al., 2020; Rahimpour et al., 2021; Deng et al., 2022; Liu et al., 2024). Our point of departure from exclusively stimulating motor targets is informed by network-level framing of PD pathophysiology. Although motor targets are a critical source of motor programs and commands, targeting regions involved in cognitive aspects of posture-gait control may better improve motor functions as well as cognitive functions. We emphasize targeting alternative connected regions of the basal ganglia-cortex-cerebellum network (BG-Ctx-CB) implicated in PD, specifically the dorsolateral prefrontal cortex (dlPFC), cerebellum (CB), and posterior parietal cortex (PPC). We suggest that targeting the BG-Ctx-CB network and using plasticity-informed stimulation paradigms will result in more consistently effective rTMS treatments for FoG motor deficits in PwP.

## A network approach to rTMS targeting

**FIGURE 1:**
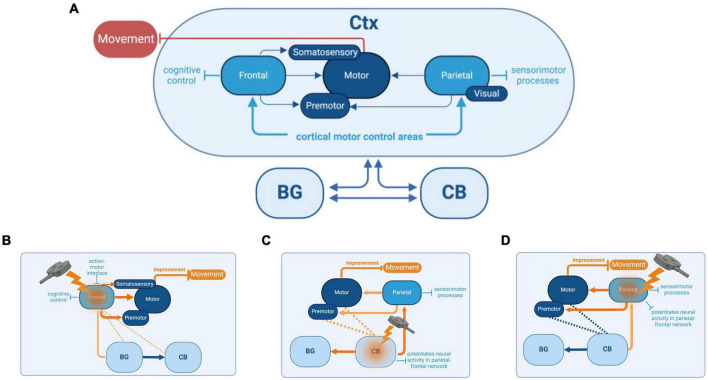
Basal Ganglia (BG)-cortex (Ctx)-cerebellum (CB) network, its functions, and the reciprocal interactions between each cortical and subcortical brain region **(A)**. Connections between BG and the thalamus, which also project to frontal, motor, and parietal cortices, as well as other downstream connections between BG and the brainstem, are not shown in this diagram (see Bohnen et al., 2022a,b, for a more comprehensive illustration of connections). Potential cognitive and sensorimotor improvements that could be achieved by targeting specific brain regions with repetitive transcranial magnetic stimulation (rTMS). The targeted regions include the frontal cortex **(B)**, cerebellum (CB) **(C)**, and parietal cortex **(D)**. The basal ganglia (BG) are also depicted for context.

PD reflects network-level disorders. BG dysfunction is the root of many motor features of PD. Focusing on BG alone, however, disregards its interactions with other regions such as dlPFC, PPC, and the CB (Figure 1A). Studies suggest PD deficits in cognitive control drive FoG, resulting from dysfunctions in a distributed network of frontal and parietal cortical regions (Rubino et al., 2014). Neuroimaging studies link cognitive symptoms in PD to changes in dlPFC, premotor, and PPC activity, and motor symptoms to changes in the interactions between BG, cortex, and CB (Owen, 2004; Michely et al., 2015; Caligiore et al., 2016, 2017).

While network interactions influencing PD features such as postural instability and FoG are well-established, most rTMS studies have focused on motor targets for treating these sensorimotor deficits. The emphasis on motor targets is due to their well-defined role in preparing and executing coordinated movements and their attractive anatomical properties. However, two-thirds of corticospinal projections originate from outside motor targets in frontal premotor areas and parietal cortices (Strick et al., 2021). Motor commands are influenced by higher-level information such as goals, attention, and context originating from other regions such as dlPFC and PPC (Gallego et al., 2022). The dlPFC and PPC have critical roles in mediating sensorimotor transformations and coordinating multifaceted aspects of movement, such as movement planning, action intentions, and decision-making (Andersen and Cui, 2009). For PwP, it is likely that deficits in the performance of these contextual and cognitive tasks influence movement and disrupt everyday motor functions (Hausdorff et al., 2006). Modulation of other regions outside of motor targets may be more effective in the treatment of PD cognitive and motor symptoms.

The choice of optimal stimulation targets should also consider person-specific compensatory mechanisms. Compensation is the brain’s adaptive response to ongoing neurodegeneration and may have beneficial or detrimental effects depending on the context and brain regions involved. As the disease progresses, certain brain regions may exhibit increased or decreased activity to offset motor and non-motor deficits (Blesa et al., 2017). This compensatory mechanism can have varying outcomes, potentially either alleviating or intensifying the symptoms and effects of PD. Understanding these compensatory mechanisms is crucial for guiding stimulation treatment strategies and targeting the appropriate brain areas to either excite beneficial compensation or inhibit harmful overactivity. In the sections that follow, we present work that has investigated brain stimulation to different nodes of BG-Ctx-CB networks involved in posture-gait control.

### Dorsolateral prefrontal cortex (dlPFC)

An alternative cortical rTMS target for improving motor impairments in PwP is the dlPFC. The dlPFC has a wide-reaching influence over a swath of executive functions (Miller and Cohen, 2001). Apart from cognitive deficits associated with PD, motor deficits may also be explained partly by dlPFC dysfunction, specifically as they relate to attentional processes (Cavanagh et al., 2022). The ability to move seamlessly through the environment depends upon the integration of sensory cues, motor commands, and cognitive demands, all filtered by attentional processes. dlPFC is instrumental in these attentional processes, exerting top-down control through projections to premotor areas, thalamus, and BG (Schall and Boucher, 2007). Cholinergic projections from the basal forebrain to dlPFC are critical for attentional integration and can be conceived as part of an Attentional-Motor Interface (AMI) network, which also includes thalamic and BG connections (Sarter et al., 2021; Albin et al., 2022). PD features such as postural instability and FoG are linked to the degradation of these cholinergic pathways (Stuart et al., 2020; Bohnen et al., 2022b). The link between motor and attentional deficits in PwP is observed in dual-tasking studies in which participants perform separate cognitive and movement tasks simultaneously (Rochester et al., 2004; Kelly et al., 2012; Salazar et al., 2017). In studies assessing both motor and cognitive effects of dual-tasking, performance declines occur in both domains (Kelly et al., 2012). For example, dual-tasking during walking often results in FOG (Nutt et al., 2011; Amboni et al., 2013; Peterson et al., 2015). This dual-task interference may be partly explained by limited attentional capacity for monitoring gait, posture, and complex movement, leading to falls due to freezing of movement, loss of balance, and inadequate rebalancing after movement errors (reviewed in Sarter et al., 2014). It is not surprising, therefore, that attentional areas influence posture-gait control (Shine et al., 2013; Zhou et al., 2021; Bardakan et al., 2022). An additional important feature of dual-task walking deficits in PwP has been associated with impairments in executive functions such as set-shifting and response inhibition (Lord et al., 2010; Plotnik et al., 2011). For instance, impairments in task switching are correlated with FoG in PwP, offering further evidence of the joint manifestations of cognitive and motor system deficits within PD (Naismith and Lewis, 2010). One possible explanation for these dual-tasking effects is that compensatory activity from the dlPFC increases in PD, leading to more top-down control of movement, and this compensatory activity is disrupted by dual-tasking that also relies on dlPFC activity. Recent evidence has demonstrated an upregulation of cholinergic neurotransmission in dlPFC of cognitively impaired early-stage PwP (Van Der Zee et al., 2022). Neuroimaging meta-analyses support this interpretation of increased dlPFC activity in PD (Stuart et al., 2018; Herz et al., 2021). It is conceivable that using excitatory rTMS to modulate the excitability of the dysfunctional dlPFC within the cognitive-motor control network could normalize its activity. This might lead to more effective neural processing for attention and executive functions related to posture and gait control, potentially alleviating FOG during secondary tasks. It is also feasible that excitatory rTMS can enhance dopamine release in the striatum, potentially replenishing depleted neural reserves and alleviating the striatal overload linked to FOG episodes (Strafella et al., 2001; Potvin-Desrochers and Paquette, 2021).

Increased dlPFC activity in PD could reflect a necessary compensatory response to ameliorate motor deficits in PD, or this excess activity could be detrimental to function. Overactivity in the dlPFC may be considered wholly detrimental rather than compensatory if the persistence of increased top-down control occurs in contexts beyond dual-tasking. If dlPFC hyperactivity is contributing to the motor deficits in PD, one treatment strategy would be to downregulate dlPFC activity through rTMS treatments. Due to its upstream and contextual influence on M1, designing rTMS paradigms for dlPFC could more effectively counteract the movement instability arising from attentional and control deficits (Figure 1B). Most treatment studies to date emphasized strengthening connections between dlPFC and M1 using excitatory rTMS, leading to mostly null effects on motor performance (Goodwill et al., 2017). Although well-intentioned, if dlPFC’s compensatory role in PD results in an excess attentional focus on the control of movement, as indexed by dual-tasking studies, future treatments that reduce dlPFC activity using inhibitory rTMS protocols may be more effective. Recent meta-analyses support this notion (Goodwill et al., 2017). Further trials are warranted to support this notion. Inhibitory rTMS protocols directed to the dlPFC are one pathway of alternative treatments that could improve motor function in PwP.

### Cerebellum (CB) and posterior parietal cortex (PPC)

Another network-level stimulation target is the CB (Figure 1C), which can modulate the neuronal excitability of interconnected PPC regions (Casula et al., 2016). The BG and CB were historically thought to interact via their common cortical outputs (Mirdamadi, 2016). Non-human primate research suggests that BG and CB are also interconnected at the subcortical levels through the subthalamic nucleus and deep cerebellar nuclei (Bostan and Strick, 2010; Bostan et al., 2013). There are dissociable cognitive and motor networks within the CB, with anterior cerebellar lobules exhibiting greater motor cortical associations, and the more posterior lobules are associated with greater cognitive cortical areas (Buckner et al., 2011; Bernard et al., 2012; Buckner, 2013). Studies investigating tremor-related activity in PD have identified cerebellar structural changes and indicated a role for cerebellar circuits, but further research using neuroimaging, brain stimulation, and consistent patient selection is required to clarify the specifics of cerebellar modulation and its impact on tremors. Concerning FoG in PD, disturbances to the BG-CB subcortical and cortical circuitry may influence the observed abnormal functionality (Carrillo et al., 2013; Bologna et al., 2015; Mirdamadi, 2016; Helmich et al., 2021). The CB is shown to play a central role in regulating both cognitive and automatic processes related to posture-gait control through its influence on the cerebral cortex (Takakusaki, 2017). Stimulating cerebellar regions offer promise in directly influencing the CB-BG-Ctx cortical circuitry, thereby improving functionality and potentially alleviating FoG symptoms in PD.

**BOX 1**

**FIGURE 2:**
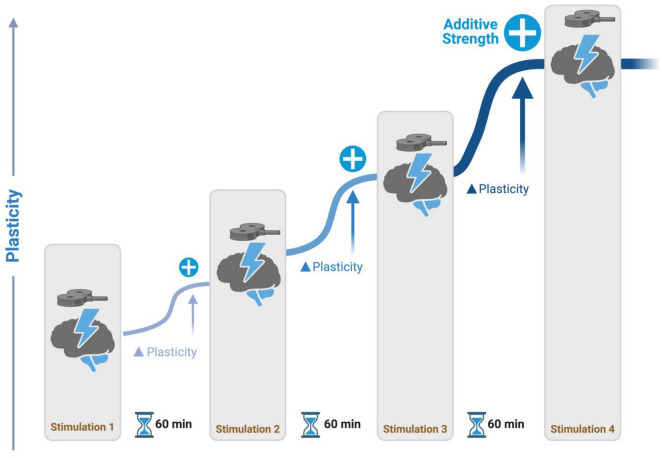
Spaced learning stimulation protocol. During stimulation, participants perform a task to activate a network of interest. Repetitive transcranial magnetic stimulation (rTMS) is delivered at intervals of 60 minutes, promoting additive potentiation to neural activity in networks.

Spaced Learning: Spaced learning involves repeated training sessions with long intervals in between and it is critical for dynamic motor memory stabilization (Overduin et al., 2006; Smolen et al., 2016). This phenomenon is due to changes in synaptic strength, where cumulative exposure to spaced stimuli promotes the formation of long-term memory traces. Metaplasticity, which refers to changes in synaptic plasticity such as long-term potentiation (LTP) or depression (LTD), has been shown to exhibit both additive and stabilizing effects. The spacing between stimulations is a key factor that determines the type of metaplasticity observed. Specifically, research indicates that increasing the timing between stimulations promotes additive metaplasticity effects (Thomson and Sack, 2020). Theta burst stimulation applied at short intervals does not produce cumulative LTP effects (Lynch et al., 2013). For example, iTBS with eight- to fifteen-minute intervals between sessions did not result in any significant differences compared to sham stimulation (Thomson et al., 2019). Animal and human studies have demonstrated that longer intervals (50 + minutes) between TBS sessions are necessary to augment LTP (Kramár et al., 2012; Lynch et al., 2013; Cao and Harris, 2014; Cole et al., 2020). These findings suggest that TBS may be a promising method for inducing additive metaplastic changes in network activity levels, thereby preventing homeostatic metaplasticity from stabilizing stimulation effects. Future research on the potential of spaced TBS to induce long-lasting neuroplasticity in the human cortex is a promising avenue to explore and can have significant implications for designing effective rehabilitation programs for PwP with balance and gait disturbances (Figure 2).

The compensatory role of CB in PD may contribute to maintaining normal motor and non-motor function in the early stages of the disease (Wu and Hallett, 2013; Bostan and Strick, 2018). Examining CB function in healthy individuals and PwP can provide valuable insights into treating motor and cognitive deficits (Rahimpour et al., 2021). PwP with greater cerebellar connectivity show higher levels of performance in motor tasks (Festini et al., 2015). Similarly, medicated PwP with cognitive and motor deficits show reduced levels of cerebellar-whole brain and within cerebellar connectivity in comparison to healthy age-matched participants when measured with resting-state fMRI (Festini et al., 2015). As past studies have done, excitatory rTMS protocols through the CB have the potential to enhance cognitive and motor pathways that are not effectively targeted by solely focusing on the M1 (Nardone et al., 2020).

Administering excitatory rTMS to the CB could enhance motor control and sensorimotor processes in the PPC. Reductions in cortical volume involving the PPC may contribute to the manifestation of FoG in PwP (Rubino et al., 2014). Although cortical volume loss is not treatable, stimulating the PPC through rTMS either directly or indirectly can promote activity (Figure 1D). Excitatory cerebellar stimulation increased PPC excitability in stroke patients who showed improved gait and balance functions and this approach could also be translated into cerebellar stimulation to encourage network-level modulation to mitigate FoG (Koch et al., 2019). Applying excitatory rTMS to the lateral CB has been seen to have an excitatory effect and influence cortical connections to the PPC in healthy individuals (Casula et al., 2016). An excitatory rTMS approach to sensorimotor regions of PPC and lateral and posterior lobules of CB (e.g., Crus I/II, lobules VI-IX) could mitigate cognitive-motor dysfunctions of FoG in PwP.

## Maximizing the effects of stimulation (frequency, dosage, rTMS-state-dependence interaction)

In addition to considering the desired treatment brain targets, treatment regimens themselves are critical when designing rTMS paradigms. Factors such as stimulation frequency, dosage, and brain state during stimulation can affect brain and behavioral responses. Although rTMS has been used in translational research to improve cognitive-motor function in PwP, mixed and weak results may be attributed to the use of suboptimal treatment regimens (Chung and Mak, 2016; Xie et al., 2021).

In clinical contexts, there are two main categories used for rTMS: standard repetitive TMS (rTMS) and patterned rTMS using theta burst stimulation (TBS). Both stimulation categories have subtypes that lead to increased cortico-spinal excitability [rTMS: > 5Hz, intermittent TBS (iTBS)] and decreased cortico-spinal excitability [rTMS: < 1Hz, continuous TBS (cTBS)] (Huang et al., 2005; Gamboa et al., 2010; McCalley et al., 2021). TBS has gained popularity in recent years due to its ability to produce the same changes in excitability as standard rTMS but in about one-tenth of the stimulation duration, and the effects last longer than those from standard rTMS (Klomjai et al., 2015; Wischnewski and Schutter, 2015). The choice of frequency should be driven by intended effects on the target location and connected networks. For example, stimulating the dlPFC with excitatory iTBS could lead to enhanced activity in other connected frontal regions. Applying iTBS to the CB would induce circuitry changes to the cortex via deep cerebellar nuclei and thalamus through complex projections. Open questions remain about the nature of the excitatory and inhibitory effects of these frequencies in areas outside of the M1 (Siebner et al., 2022). One possible approach involves using fMRI to establish the relationship between stimulation and functional connectivity and between stimulation and FoG. This allows for the optimization of rTMS protocols, which can be used to develop effective therapeutic interventions based on idealized brain network function mediating behavior.

Another important variable in defining rTMS treatment paradigms for PD is stimulation dosage. “Dosage” is defined as the number of pulses administered in one treatment day. One possible explanation for the heterogeneity of outcomes in the use of rTMS for motor symptom treatment in PwP is inadequate dosing of stimulation. Most studies focusing on motor symptoms in PwP have been limited to small dosages of around 2,000 pulses and short treatment durations between one and three days. The most effective PD treatment studies so far have been those that greatly increased the dosage and duration of the treatment course, with approximately 10,000 pulses delivered over weeks (Chou et al., 2015). Inadequate rTMS dosing was similarly proposed to be an issue for the use of rTMS as a treatment for depression (Hutton et al., 2023). New treatment regimens such as the Stanford Accelerated Intelligent Neuromodulation Therapy (SAINT) demonstrated the safety, tolerability, and enhanced clinical effectiveness of an accelerated, high-dose iTBS treatment paradigm (Cole et al., 2020). The SAINT protocol demonstrates the promise of incorporating spaced learning principles for the timing between sessions to enhance rTMS treatments’ effectiveness (see Box 1). Additional research is necessary to determine the appropriate dosage for neuromodulatory therapies aimed at alleviating FoG symptoms in PwP.

The behavioral and neural activity, referred to as ‘brain state’, during stimulation is another often overlooked factor that may be critical for designing effective rTMS therapies. The large inter- and intra-individual variability of brain and behavior responses to rTMS therapies in PD could be reduced by controlling the brain state at the time of stimulation. Current rTMS interventions are often administered during a brain state termed ‘rest’ when the participant is in a quiet, still position (Bradley et al., 2022). One potential difference in treatment outcome may arise from variations in the ‘rest’ brain state between individuals. If the goal is to improve movement, it may be more effective to deliver stimulation while the brain networks responsible for generating movement are engaged (Cattaneo and Silvanto, 2008; Silvanto and Cattaneo, 2014; Pitcher et al., 2021; Bradley et al., 2022; Goldenkoff et al., 2023). Controlling the behavioral state, such as by asking participants to perform a goal-directed behavior, could reduce neural activity fluctuations by constraining the pattern of interactions between brain regions engaged in cognitive-motor control. Administering brain stimulation adaptively when the individual generates a motor response might not only produce distinct effects on long-range connectivity in the engaged network during stimulation but also lead to marked modulators of plasticity mediating cognitive-motor control.

The studies reviewed here demonstrate the frequency, dose, and state-dependent effects of rTMS on brain activity and behavior. The consequences of repeated stimulation in such circumstances have not been fully explored. Future research should consider increasing the dosage of rTMS and delivering distinct stimulation frequencies based on ongoing physiology to design more effective treatments for cognitive-motor impairments in PwP. Additionally, matching the behavioral state during stimulation to target specific neuronal populations involved in cognitive-motor control may provide another avenue to reduce heterogeneity and increase the efficacy of neuromodulation treatments in PD. Understanding the frequency, dose, and state-dependent effects of neural stimulation is critical to improving clinical outcomes by identifying the factors that affect response to rTMS.

## Conclusion and future directions

In this opinion piece, we have outlined how taking a network view of PD may lead to selecting more effective rTMS targets for alleviating postural imbalances and FoG in PwP. While rTMS to motor targets has been a standard intervention, recent insights suggest that targeting a node through specific brain circuits involved in cognitive-motor control, such as dlPFC, CB, and PPC, could increase the effectiveness of neuromodulation of aberrant brain networks in PD. This network approach also raises the prospect of delivering multi-site rTMS concurrently to modify connectivity in brain networks in clinical contexts. The precise interactions between brain regions and stimulation are complex and require a causal grounding of clinical neurostimulation in specific neural circuit alteration. Emphasis should be placed on neural mechanisms of treatment using neurophysiology and neuroimaging to increase the reliability of these interventions. Additionally, choosing the correct frequency, increasing the rTMS dosage, and integrating brain state manipulations into stimulation sessions may provide more effective and lasting changes than currently demonstrated. Future work should focus on which rTMS parameters, such as iTBS or cTBS, would be most effective at improving symptoms at the different targets. To fully harness the therapeutic potential of rTMS in PwP, it is crucial to gather data across all disease stages, identify optimal stimulation timing within the disease course, and determine the duration of stimulation effects on brain activity and behavior outcomes. By evaluating factors such as medication status and disease progression, we can develop an adaptive, individualized stimulation protocol for each patient, maximizing therapeutic outcomes and quality of life while maintaining pragmatic treatment goals.

## Data Availability

The original contributions presented in the study are included in the article/supplementary material, further inquiries can be directed to the corresponding author.
